# Determinants affecting medication adherence in the elderly: A qualitative study

**DOI:** 10.1002/agm2.12132

**Published:** 2020-11-10

**Authors:** Peivand Bastani, Parisa Bikineh, Ramin Ravangard, Rita Rezaee, Zahra Kavosi

**Affiliations:** ^1^ Health Human Resources Research Center School of Management and Medical Informatics Shiraz University of Medical Sciences Shiraz Iran; ^2^ Student Research Committee Shiraz University of Medical Sciences Shiraz Iran

**Keywords:** elderly, medication adherence, qualitative study

## Abstract

**Objective:**

Due to high rates of irrational use of medicines and low rates of medication adherence among older Iranian patients, this study aimed to explore the determinants of medication adherence in the elderly.

**Methods:**

This qualitative study was conducted in 2019 via 20 semi‐structured interviews with a purposeful sample of elderly people and health‐care personnel. Transcript data were analyzed applying thematic analysis. MAXQDA_10_ was used for thematic analysis and presenting a thematic map.

**Results:**

Two main themes appeared as the main determinants of medication adherence: individual determinants and external determinants. Other results show that there were six sub‐themes and 23 main categories in this regard. The sub‐themes were as follows: consumption disorder, consumption acceptance, delinquency, forgetfulness, sociocultural factors, and others’ impacts.

**Conclusions:**

Different individual and external factors can affect elderly people’s medication adherence. Considering the significance of this issue for the health of elderly people and the whole community, positive changes are needed to manage the medication‐adherence process. Basic and scientific planning, appropriate interventions, and comprehensive collaboration among all health‐system levels are required for implementing such changes.

## Graphical abstract

Individual factors the same as medicine consumption disorders, forgetfulness and delinquency can affect the elderly`s medication adherence negatively. However, medicine consumption acceptance by the elderly can positively influence the adherence. These are along with the external factors as well as sociocultural ones.

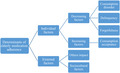

## 1 INTRODUCTION

Nowadays, different communities have reached higher average ages with the aid of socioeconomic development and continuous growth of citizens’ life expectancies.^1^ The population’s elderly is a relatively new global health issue that is caused by different factors, such as decreased birth rates, medical technology improvements, public health and education development, and the global increase of life expectancy.^2^

At an individual level, old age is a dominant biologic characteristic that follows different symptoms, such as changes in the face and the whole body, hair color, and other vital and physiologic organs. According to the definition of the World Health Organization (WHO), old age is considered as the period of life that starts from the end of 60 years and the beginning of 61 years.^3^ The process of population aging is considered as a multidisciplinary issue, with humanistic, socioeconomic, cultural, and health dimensions.^4^ The population’s transition and aging are considered as one of the main challenges for health‐care providers in the 21st Century.^5^ According to the statistics, the global population increases by almost 1.7% annually and the population older than 65 years increases by 2.5%.^6^ The aging population has resulted in great changes in the prevalence of diseases, from a significant reduction in contagious illnesses to higher life expectancies and increases in chronic diseases. This change is known as the silent revolution and has led to more attention for elderly health concepts and the elderly’s quality of life.^7^

For many reasons, elderly people are vulnerable to problems related to medicine consumption.^8^ In this regard, the rational use of medicines has been an important consideration for the WHO and for ministries of health worldwide. The rational use of medicines is an important issue for the entire population, as well as the elderly, and is related to a community’s cultural, social, and sometimes religious context. Irrational medicine consumption can lead to an increased mortality rate, disability, and great costs because of the pharmaceuticals’ side‐effects.^9^ This issue is crucial for the elderly, considering their physical and psychological status and their important roles as family and community members.^10^

As a low‐income developing country in the Middle East, Iran is experiencing an increase in the older population. Such a transition is accompanied by new risk factors for this population, with its particular medical and pharmaceutical needs.^11^ At the same time, evidence indicates a higher prevalence of irrational use of medicine and self‐medication among the Iranian community than in other countries.^12^ This evidence, along with the low overall prevalence of medication adherence in Iran^13^ and particularly the low medication adherence among elderly people in the center of Iran,^14^ make this issue significant.

According to these factors, this study was conducted to explore the main determinants of medication adherence among the elderly Iranian population.

## 2 METHODS

This study was carried out in 2019 as a qualitative research applying a content‐analysis method. Data were collected via 20 semi‐structured interviews with the aim of exploring the main determinants of medication adherence in the elderly. Study participants included six health‐care personnel (physicians, nurses, and pharmacists) and 14 participants aged older than 60 years. These participants were selected through purposeful and convenience sampling. The inclusion criteria were having enough information and knowledge or related experience about the topic, and being talkative and willing to share the information via interviews. All the interviews were conducted face‐to‐face and individually after participants had provided permission. At the beginning of the interview sessions, general explanations were presented about the aims of the study and the ethical issues, as well as commitment for confidentiality of the data, either as audio or text. Following these explanations, participants were asked to sign the written voluntary consent and were assured that they could quit the interview or stop talking whenever they wanted. The study was approved by the Shiraz University of Medical Sciences Ethics Committee (ID IR.SUMS.REC.1398.541).

The interview topic guide contained eight main questions about the determinants of medication adherence according to the elderly participants’ experiences or the personnel’s points of view. Then, according to their answers, the number of questions could be increased to 16 and 13 in the elderly and personnel groups, respectively. The average of the interview sessions varied from 55 ± 15 minutes for the elderly participants to 45 ± 20 minutes for the health‐care personnel. All the interviews were conducted by one of the researchers (P. B.). All of the interview sessions were recorded with the participant’s approval and word‐for‐word transcripts were made from all of the audio recordings immediately after the interviews. During interviews, the interviewer noted the most important details and these points were confirmed by the participants at the end of each session. As mentioned, during the interview sessions we sought the main factors and determinants affecting medication adherence based on the personal experiences of the elderly participants and the viewpoints of the personnel, who visit and work with the elderly regularly. The interviews were continued to achieve a saturation level and after 20 interviews, the data were saturated, and the interview process was stopped.

In order to increase the validity and robustness of the qualitative study, the four criteria of credibility, transformability, dependability, and confirmability proposed by Guba and Lincoln were applied.^15^ In order to achieve credibility, long‐term engagement, continuous comparison of the data, and their categorization were used. To reach confirmability, the process of data coding was reviewed, and the related content of the interviews was shared with some experts in qualitative studies. For more dependability, the trial audit was conducted through the members of the research team and the experts as well and, finally, in order to assure transformability, a comprehensive description of the concept, description of the participants’ characteristics, and a complete clarification of data collection and data analysis were used along with the examples of participants’ statements.

In order to analyze the data, the approach proposed by Graneheim and Lundman was used.^16^ In this regard, all the audio recordings were listened to several times for familiarity with the data and the transcripts were written many times. The most meaningful units of text were extracted, considering the aim of the study, and the initial codes were made and labeled. Then, the initial codes were reviewed and refined to obtain the final codes. In the second step, the final codes were categorized to reveal sub‐themes and main themes. This process was conducted by two of the researchers separately in order to increase the consistency of the data analysis. Cases of different coding were discussed by the research team to achieve an agreement. At the end of this process, the main determinants of medication adherence in the elderly were explored. MAXQDA_10_ was applied to analyze the data.

## 3 RESULTS

Table 1 shows the characteristics of the study participants. Elderly participants were aged 62‐74 years; and health‐care personnel had been employed for 12‐30 years. Most of the participants in the elderly group were female (71.4%).

**Table 1:** Characteristics of the study participants

Participants category	Age/years employed		Gender	Occupation	Education level
Health‐care personnel	Years employed	30	Male	Pharmacist	PharmD
		12	Male	Physician	MD
		20	Male	Physician	MD
		22	Female	Nurse	MSc
		25	Female	Nurse	MSc
		30	Female	Health programmer	MSc
Elderly participants	Age (years)	65	Female	Housewife	Diploma
		69	Male	Retired technician	BSc
		67	Male	Constructor	BSc
		60	Female	Housewife	Less than high school
		62	Female	Retired teacher	Diploma
		72	Female	Housewife	Less than high school
		67	Female	Retired clerk	Diploma
		74	Male	Retired clerk	BSc
		71	Female	Housewife	Diploma
		70	Female	Housewife	Less than high school
		60	Female	Housewife	Diploma
		67	Female	Housewife	Less than high school
		64	Male	Retired clerk	Associate
		62	Female	Retired clerk	Diploma

Results of the content analysis indicate that two main themes appeared as the main determinants of medication adherence: individual determinants and external determinants. Other results show that there were six sub‐themes and 23 main categories in this regard. The sub‐themes were as follows: consumption disorder, consumption acceptance, delinquency, forgetfulness, sociocultural factors, and others’ impacts. The initial four sub‐themes were related to individual determinants, while the sociocultural factors and others’ impacts were categorized as external determinants (Table 2).

**Table 2:** Main determinants of medication adherence in the elderly

Main themes	Sub‐themes	Main category
Individual determinants	Consumption disorder	Unwillingness and doubtfulness
		Complicated instructions
		Side‐effects
		Lifestyle
		Unawareness
		Psychological condition
	Consumption acceptance	Trust in physician
		Fear of complications
		Social load and problems
		Strong belief in consumption
	Delinquency	Personalized consumption
		Personalized prescription
		Without consulting use
		Ignoring
		Individual characteristics
	Forgetfulness	Age
		Special conditions
External determinants	Sociocultural factors	Culture
		Literacy
		Education and knowledge
		Health‐system context
		Insurance system
	Others’ impacts	Providers/caregivers
		Relatives

As shown in Table 2, the determinants of medication adherence could be divided into two main determinants: individual and external. In the individual determinant, the core issue is the elderly person’s behavior in regard to medication consumption; while in the external determinant, the impacts of others on the elderly people are considered. Each of these determinants is clarified and exemplified below.

### 3.1 Individual determinants

Individual determinants are directly related to the elderly people and their behaviors. Although they have the same nature, they can be categorized into two different factors of reinforcing and preventing factors of medication adherence. The related sub‐themes are presented below.

#### 3.1.1 Consumption disorder

Consumption of different medicines can be stressful and can involve various sets of instructions. Such situations can lead to errors in the time, type, or dose of consumption, and can also cause side‐effects due to an elderly patient’s lifestyle change, physical or psychological condition, misunderstanding, or lack of knowledge. All of these factors can decisively lead to a *disorder* or discomfort in medicine consumption. In this regard, one of the participants said:

Medication consumption disorder is really important. We give them their pharmaceuticals and when they go home, the problems start. They may take the night medicine during the day or reverse. This is particularly important for anti‐hypertensions and diuretics that should be taken in the day. [P3]

#### 3.1.2 Delinquency

Personalized behaviors, as reported by the elderly, the physicians, or other caregivers, can lead to many problems. In addition, use of medicines by the elderly that is irrational or without consultation, and failure to complete the medicine’s duration for any reason can easily lead to medication delinquency. One of the participants declared:

Sometimes they come and want their medicines without prescription or according to their last prescriptions. This usually occurs for sedatives, such as alprazolam or zolpidem. [P1]

Another participant stated:

Many of them cut their medicine dosage suddenly and whenever they feel a little problem, even though the physicians emphasize the gradual stop of the medicine. This can clearly lead to different side‐effects and may worsen the condition of the elderly person. [P6]

#### 3.1.3 Forgetfulness

One of the main reasons for lack of medication adherence in elderly people is forgetfulness and the conditions that cause it. Forgetfulness can be considered in regard to three aspects: First, aging can normally cause physical and psychological changes, such as decrease in awareness level, Alzheimer’s disease, and neglect of one’s health. Second, a particular situation, such as a vacation, loneliness, or lack of a guardian or companion can result in forgetfulness. Finally, complicated medicine instructions can cause confusion and forgetfulness. In this regard, one of the elderly participants said:

I should take hypertension medicines every day, but many days I forget to take them. It has many reasons: my busy and troubled days, being in a hurry and so on. Then I feel headache and I remember that the medicines are lost. [P11]

#### 3.1.4 Consumption acceptance

Trust in the physician’s orders, positive beliefs in the routine consumption of medicines, the elderly patient’s degree of dependence on his/her family members, and anxiety and fear of complications because of inappropriate or untimely use of medicines are among the factors that can lead to elderly people’s consumption acceptance of medicines. In this regard, one of the elderly participants indicated:

When I feel better, I have a strange tendency to stop my medicines … but right after I become regretful. [P8]

Another said:

Now my family force me to continue my medicines. I feel tired of these many pills I should take every day. [P19]

### 3.2 External determinants

The second main theme as a main determinant of medication adherence in the elderly involved nonindividual factors with external reasons. Although these factors do not depend on the elderly person directly, their changes or quality can affect the adherence of medications among elderly people indirectly. Ignoring or reinforcing these factors can make positive or negative effects on the process of medication adherence in the elderly. Its related sub‐themes are described below.

#### 3.2.1 Sociocultural factors

Many factors can affect the adherence or nonadherence of elderly people in regard to their daily prescribed pharmaceuticals, such as their cultural status, literacy level, education, past learnings, the whole community’s level of information and knowledge as well as that of elderly people, and the country’s insurance and health system. In this regard one of the elderly participants claimed:

The culture of family is important; our history [and] whatever we have learnt from our parents are really effective. For example, when my father used to drink herbal teas to be relaxed, it transferred to me and I prefer to do so, instead of taking pills. [P16]

Another participant noted:

Our policy‐makers sometimes have conflicts of interest; ie, sometimes they play two different roles as a policy‐maker and at the same time, the stakeholder of a pharmaceutical company. Such a conflict of interest can prevent them conducting any effective intervention and leads to supply‐induced demand or medicine overuse. These are inevitable social forces. [P3]

#### 3.2.2 Others’ impacts

The role of literacy and skills of the practitioners in diagnosis and prescription can lead to appropriate distribution and delivery of the medicine by the pharmacies. This factor, along with the impact of social networks, relatives, and the family, can result in preparation and consumption of the required medicines for elderly patients and determine the quality of their care. In this regard, the present participants had important statements, as follows:

A physician should give enough description to the elderly person about his/her health condition, the necessary care and the medicines. If the elderly person is not aware or cannot remember the important tips, the physician should inform his/her companion if possible, so that the family members or guardian know the time and instructions of medication consumption. [P5]

Also:

Sometimes I take my medicine but when I get home my sons or the other relatives call and want me to stop the ordered medicine and use herbal combinations or food instead. That’s why I cannot finish the duration of my treatment. [P15]

**Figure 1:**
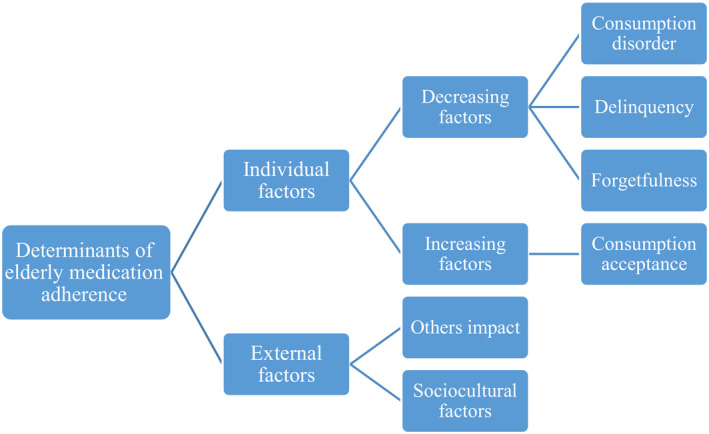
Thematic map of determinants affecting medication adherence in the elderly.

Figure 1 clearly illustrates the above relations among the main and sub‐determinants of medication adherence in the elderly.

## 4 DISCUSSION

According to our results, consumption disorder, delinquency, and forgetfulness are among the preventing and decreasing factors of medication adherence in the elderly. In contrast, consumption acceptance is considered as an increasing factor for the elderly’s medication adherence. Similarly, different studies have emphasized various reasons for lack of medication adherence. Among them, we can consider forgetfulness, lack of incentive, high cost of medicines, inadequate health literacy, insufficient or complicated medication regimen, lack of trust in practitioners, neglect, side‐effects of medicines, religious considerations, and misunderstanding of the prescriber’s orders.^17^, ^18^

Other studies have mentioned some facilitators for medication adherence, including reduction of inappropriate and complicated prescriptions, support from health‐care team members, improvement of the patient‐physician relationship quality, increased sense of trust, and innovation in medicine instructions for the elderly.^19^, ^20^ Moreover, the WHO has presented a framework for medication adherence and has categorized the important factors into five categories as follows: socioeconomic factors, factors related to the health‐care team and service delivery, factors related to treatment, factors associated with the illness, and finally factors related to the patient.^21^ Although many of these factors are unchangeable, some can be changed with patient education and improving patients’ knowledge and attitudes. Other interventions can be applied to help patients overcome their forgetfulness, such as providing movable brochures about medication times or instructions to be pasted on the refrigerator or sending reminder messages. Also, patients can be advised to prepare appropriate boxes with their medicines to decrease the risk of forgetfulness.

According to Salzman, lack of medication adherence can include intentional or unintentional overuse of medicines and mistakes in the time and method of consumption.^22^ In this regard, the present results indicating patients’ beliefs that a greater use of medicines results in a faster cure exemplify a lack of medication adherence. Other causes of forgetfulness as a strong individual determinant can affect the elderly’s medication adherence. Jaam et al also focused on six main determinants for patients’ behavior toward medication adherence, consisting of the patients themselves, the health‐care providers, the health‐care system, the community, the illness, and the medicines.^23^ These findings all indicate the roles of the individual and of external determinants, like the present results.

The other results of our study show that two factors—sociocultural and others’ impacts—can be considered as the main external determinants affecting medication adherence in the elderly. In this regard, Murray et al have mentioned the external environment, including a combination of the elderly person’s home and society, and the level of support related to these resources as the main factors of medication adherence.^24^ Holt et al have also emphasized the importance of an open patient‐physician relationship as an important facilitator in the process of medication adherence. The cost and coverage of medicines were also mentioned as significant barriers in the policy‐making level. Moreover, interpersonal relationships, particularly family support, were considered as important factors in commitment for medication adherence.^25^

Another important factor mentioned in other studies is the relationship between patients and health‐care providers.^26^ In some situations, a patient may act dishonestly where they fail to follow the physician’s recommendations, but lie about it due to fear of the physician’s anger on similar reasons. At the same time, in another context, inadequate acceptance of health‐care providers, such as family physicians, may lead to self‐medication by patients.^27^ Thus, the relationship between patients and their caregivers should be nurtured, particularly between physicians and older patients.

### 4.1 Conclusions

According to the results, different individual and external factors can affect elderly people’s medication adherence. Considering the significance of this issue for the health of elderly people and the whole community, positive changes are needed to manage the medication‐adherence process. Basic and scientific planning is the first requirement of this management. At the same time, appropriate interventions and a comprehensive collaboration among all health‐system levels are required for implementing such changes.

### 4.2 Limitations

The current study involved some limitations. According to the multidisciplinary nature of health care for the elderly and the complications related to this concept, complementary studies are needed to determine the impacts of each of the six present determinants in depth and to shed light for policy‐makers for their further planning. At the same time, like other qualitative studies, the results can be justified according to the sociocultural context and they are not generalizable to different settings.

## ACKNOWLEDGMENTS

This study was supported by Shiraz University of Medical Sciences (97‐01‐07‐19309).

## CONFLICTS OF INTEREST

Nothing to disclose.

## AUTHOR CONTRIBUTIONS

All the authors have seen and confirmed the final version of the manuscript.
